# Statins slash CVD risk in people with HIV: global study

**Published:** 2023

A recent study suggests that one daily statin reduces the risk of heart disease in younger people with HIV, this group having a 1.5 to two times higher risk of developing cardiovascular disease than the rest of the population, say the researchers.

‘What the study is saying is if you add a statin treatment to antiretroviral therapy … that will now prevent, to a large degree, the excess cardiovascular risk associated with HIV,’ said Steven Grinspoon, lead author and professor of medicine at Harvard Medical School.

The tools currently used to make this determination underestimate the risk in people with HIV, and as a result, while existing treatment guidelines recognise that HIV increases the risk of cardiovascular disease, there has, until now, been no evidence that providing treatment was effective. The results from the latest study could potentially lead to updated treatment guidelines for reducing cardiovascular risk in people with HIV, added the scientists.

STAT News reports that the study, called REPRIEVE, is the first to globally assess a way to prevent major adverse cardiovascular events, such as heart attack and stroke, in people with HIV. The phase three trial involved 7 769 participants aged 40 to 75 years, about a third of whom were women and about 65% non-white.

Researchers hypothesised that using a statin would both lower low-density lipoprotein cholesterol and reduce inflammation: they were surprised, however, that participants who took pitavastatin lowered their risk of major adverse cardiovascular events by 35% compared with those taking a placebo – they had expected only a 30% reduction.

The randomised, double-blind trial was conducted in 12 countries in Asia, Europe, North America, South America and Africa. This is important because this study reflects the 37 million people who live with HIV globally, by including important sub-groups that are typically not reflected in large studies.

‘Often, studies don’t have enough women to make a determination or are not extended to sub-Saharan Africa or other countries,’ said Grinspoon, who is also chief of the metabolism unit at Massachusetts General Hospital. ‘So this study being global is really important because this is not just a result in North America.’

The results indicate that statins were equally effective in preventing heart attack and stroke – consistent with results from a previous study called JUPITER, which focused on slightly older patients, with an average age of 66 years, not infected with HIV.

In the past, clinicians would address lifestyle changes or do watchful waiting before prescribing a statin for patients with low to moderate risk. But now, Grinspoon said, he would consider prescribing a statin based on these new findings.

Given prior evidence of the effectiveness of statins in reducing cardiovascular risk, the results from the trial were not a surprise, said Matthew Feinstein, a physician-scientist at Northwestern Medicine and an assistant professor of cardiology at the Northwestern University Feinberg School of Medicine, who was not involved in the study.

He found two things surprising, he added. ‘The first was how big the benefit was, and two, that it extended in a somewhat lower-risk population, at least by traditional cardiovascular risk assessments.’

Feinstein added that doctors would also extrapolate data from previous trials, such as JUPITER, to help guide treatment, but these new clinical endpoints provide additional guidance for the medical community. However, he added, there are unanswered questions, including if a high-intensity statin would result in an even larger relative risk reduction, and if there would be targeted therapies developed to tackle immune response and inflammation, and potentially lower heart disease risk.

Beyond HIV, Grinspoon hopes future analysis of the data collected will help address other inflammatory diseases associated with increased cardiovascular risk, such ase psoriasis and lupus.
